# Design and Development of Virtual Reality Application Implementing Age-Friendly Care and the 4Ms: A Quality Improvement Project

**DOI:** 10.3390/ijerph21101279

**Published:** 2024-09-25

**Authors:** Sweta Tewary, Oksana Shnayder, Marie Dezine, Naushira Pandya

**Affiliations:** 1 Department of Geriatrics, Dr. Kiran C. Patel College of Osteopathic Medicine, Nova Southeastern University, Development and Implementation Age Friendly Care and 4Ms through Virtual Reality, Fort Lauderdale, FL 33328, USA; pandya@nova.edu; 2 South Florida Geriatric Workforce Enhancement Program, Department of Geriatrics, Dr. Kiran C. Patel College of Osteopathic Medicine, Nova Southeastern University, Fort Lauderdale, FL 33328, USA; oshnayde@nova.edu (O.S.); dezinemarie0@gmail.com (M.D.)

**Keywords:** virtual reality, Age-Friendly Health Systems, medical education, older adults

## Abstract

Introduction: With an increase in the aging population, the application of evidence-based practice in geriatric education can strengthen knowledge, skills, and clinical experience for healthcare students caring for older adults. The Age-Friendly Health System (AFHS) is one of the frameworks founded on providing evidence-based and low-risk care centered on what matters most to older adults, their families, and caregivers. Virtual reality (VR) platforms are gaining popularity due to their ability to provide an immersive, hands-on learning experience resembling an actual medical practice or care setting. Immersive learning enhances students’ sensory perceptions, promoting an innovative and engaging way of acquiring concepts that are difficult to teach in real life. This study aimed to design, develop, implement, and evaluate a case-based training module highlighting AFHS and educating medical students on the 4Ms approach in geriatric care (What Matters, Medication, Mentation, and Mobility). Methodology: The project was a feasibility study completed in two phases. Phase one included planning and developing a case-based scenario incorporating the 4Ms of AFHS. Phase two included implementing and evaluating the VR training module into the geriatric curriculum for medical students. Results: The final VR case displays a hospital and post-acute setting where an elderly patient is admitted for a hip fracture. Students learn how to triage and treat patients from admission to discharge while demonstrating their knowledge of AFHSs. Approximately 10% of students completed the evaluation survey, and preliminary results indicate significant knowledge change in pre-post scenario-based training on an AFHS. Conclusions: The VR education platform and embedded scenario promise an innovative adaptation of technology in learning the concepts of the 4Ms of AFHSs. However, future studies should explore VR education with clinical assessment evaluation to ensure competence in providing age-friendly care.

## 1. Introduction

### 1.1. Age-Friendly Health Systems and Geriatric Care

There is a growing need for improved competency and practice change for the healthcare workforce in providing care to older adults with complex needs. In the US, nearly half of older adults have four or more chronic conditions, and addressing all health needs of patients with multiple comorbidities during primary care visits within current practice guidelines may be challenging [1]. The Age-Friendly Health System (AFHS) is a practice framework based on providing evidence-based, low-risk, coordinated care centered on what matters most to older adults, their families, and caregivers. This practice approach uses the 4M model (What Matters, Medication, Mentation, and Mobility) on patient-centered care [2]. An AFHS helps promote overall care for older adults at every point of care, ranging from primary care, hospitalizations, and skilled rehabilitation facilities to assisted living facilities [3]. The main goal of AFHSs is to maintain a healthy, safe, and improved quality of life in older adult patients by proactively providing essential care, timely deprescribing of or avoiding unnecessary medications, supporting mental health, and managing end-of-life issues [4].

We have partially successfully implemented the 4Ms in an academic setting in a geriatric medicine clinic and have Level 2 certification through the Institute of Health Care Improvement (IHI); however, AFHS transformation is a complex process and requires participation at multiple levels. These include the organization, provider, staff, and patient participation. Consistent education for physicians, residents, fellows, and students provides ongoing reinforcement within the organizations to improve many e-clinical measures successfully. Practicing physicians lack education regarding the benefits of providing 4Ms care across the care continuum. The challenge of implementing medication reconciliation, mentation screenings, assessing fall risk for every older adult over 65, discussing health-related goals, and advancing directives is still a struggle.

### 1.2. Innovations in Medical Education

The COVID-19 pandemic expedited the digital transformation of the education sector by expanding distance learning trends and advancing innovations in educational technologies [5]. The pandemic has forced educators to provide teaching outside the traditional curriculum and adopt new education delivery methods, including e-learning [6]. With didactic education still being the driving force to educate medical students, innovative teaching methods are included in the medical education curriculum to improve students’ attention and practice competency. Advances in recent technology have helped shape education and practice in rural and underserved areas to meet the educational needs of healthcare providers. 

The complexity of the healthcare needs of an aging population further enforces the need to educate future clinicians through innovative clinical learning experiences and interprofessional approaches in medical education [7]. Virtual Reality (VR) in medical education has been evolving over the past several decades, and its usage is expected to grow by USD 143.3 billion during 2021–2025 [8,9]. VR simulations are being progressively introduced across medical and nursing schools as an effective method of delivering education [7]. The use of immersive technology as an educational tool has improved learning outcomes for healthcare students [10]. 

Additionally, incorporating VR training in the first two years of medical education allows students to improve their clinical skills and gain competency while working with patients during their clinical rotations [11]. Compared with traditional clinical education, VR simulations have proven more effective in both immediate and long-term education outcomes [12]. 

Medical education offers several hours for clinical training in geriatrics, and it is essential that 4Ms training is included in the curriculum to advance practice change in geriatrics, and it is important that 4Ms training is included in the curriculum to advance practice change for future clinicians. We propose the project planning, curriculum development, curriculum implementation, and evaluation of a VR curriculum with hands-on training that captures an evidence-based approach based on AFHS to care for older adults. By working with our medical providers and students to enhance education in both classroom and hands-on experiences, we will integrate learning opportunities throughout the 4-year medical education program. The objectives of the paper are to describe (1) planning and developing a virtual reality case; (2) implementing the VR case to medical students through geriatric curriculum; and (3) comparing changes in knowledge toward AFHS before and after completing the case-based virtual reality. 

## 2. Methods

Phase 1. Planning and development of AFHS curriculum: The study planning phase required purchasing and training Alienware software and an Oculus Rift headset. The Acadicus software was directly downloaded from their website with a secure user ID and password. The development process included a detailed design document populating the case through Acadicus scenes with prop assets, interactive elements, character assets, integrated quiz boards, playable 3D recordings (holocrons), scene change buttons, and the student starting location. The different scenarios and healthcare settings were specifically developed to provide students with diverse clinical experience and address each of the 4Ms. The educational content of the AFHS training module was based on a unique case of a geriatric patient (Millie). It included clinical and interactive components developed by a team of experts, including geriatricians, faculty, and biomedical informatics technology specialists. The module includes audio narration by a senior geriatrician to help navigate students through the course and various settings.

Phase 2: Implementation and evaluation of the VR training module. Following the module development, we conceptualized the implementation process of the case-based AFHS training in medical students’ curriculum. Since the geriatrics elective was offered for third- and fourth-year students in medical school, we modified the geriatric curriculum to incorporate AFHS education in medical school’s third and fourth years. 

AFHS training was delivered to approximately 400 medical students via different modalities: (1) virtual reality simulation (students used the VR equipment in the campus lab settings), (2) non-VR mode (students connected to the Acadicus platform and were able to navigate through the scenes and use interactive tools to complete training session), and (3) a recorded YouTube training video (option available for interprofessional education and independent distant learning).

Upon completion of the training, all students were asked to answer a self-administered short knowledge survey, which consisted of multiple-choice questions related to knowledge improvement developed by the SFGWEP team. Data were entered into RedCap, a mature, secure web application for building and managing online surveys and databases. Descriptive data, including frequencies and percentages, were used to summarize and describe the data collected through feedback surveys. A post design using the Wilcoxon signed-ranks test was used to analyze the data through SPSS Statistics (IBM, Armonk, NY, USA) version 29 for Windows. Statistical significance was set at a *p*-value of <0.05. IRB approval was waived for this study due to the nature of the study. The study was a quality improvement project and nonhuman subject research; IRB # 2023-282. The protocol does not require IRB review or approval because its procedures do not fall within IRB’s jurisdiction based on 45 CFR 46.102. Therefore, the protocol has been classified as “Research outside the purview of the IRB” for IRB purposes; this study may still be classified as “research” for academic purposes or for other regulations, such as regulations pertaining to educational records (FERPA) and/or protected health information (HIPAA).

## 3. Results

**Figure 1:**
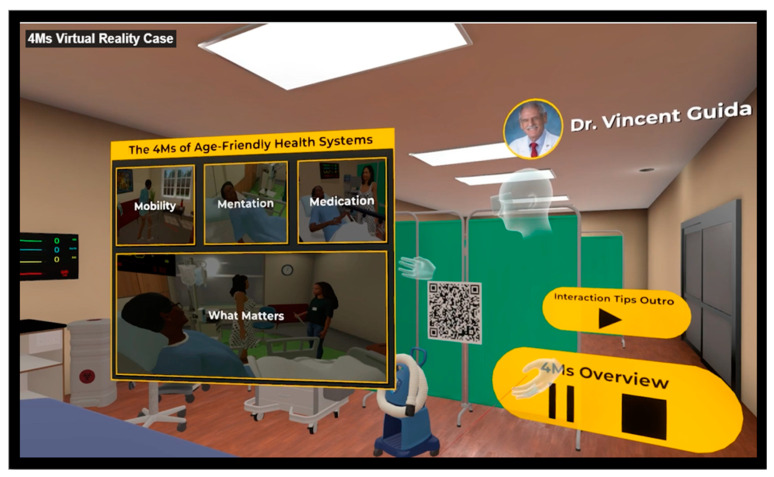
Virtual Reality Scenario.

**Figure 2:**
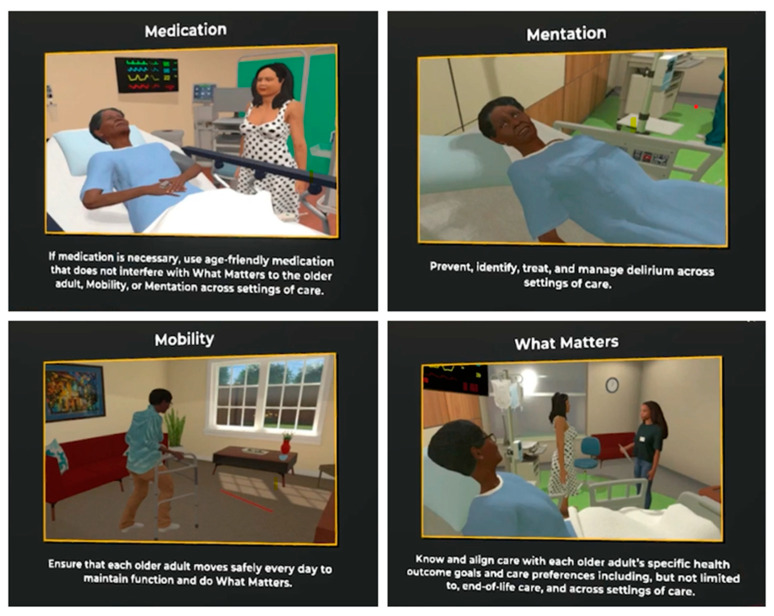
Phase 2: Implementation and evaluation of the VR training module.

Phase 1. Planning and development of AFHS curriculum: Laptops and headsets were expensive to purchase, maintain, and store in a safe place. Training faculty and staff in using virtual reality was challenging and time-consuming. Development of the case scenario and the user design document required almost 20 h of faculty time, which involved several group meetings and editing the “script” for the developers. The case was written and edited from an AFHS perspective, detailing all the 4Ms necessary for medical students to learn and apply in their clinical practice. The published case introduces students to several situations in which an older adult named “Millie”, who is an 87-year-old female, is presented with a history of hypertension, diabetes mellitus, diabetic peripheral neuropathy, urinary incontinence, chronic kidney disease, osteoarthritis, and macular degeneration. She fell while going to the bathroom at night and pressed the emergency alert button. The scenario illustrates her healthcare journey and describes the importance of aligning care with an older adult’s preferences and health outcome goals to help students identify strategies for incorporating the 4Ms framework (What Matters, Medication, Mentation, and Mobility) into a healthcare setting for older adults. (See Figure 1 and Figure 2).

Phase 2: Students who chose geriatrics as an elective rotation were provided hands-on experience implementing the 4Ms through the interactive case scenario. In stage 1, students navigate through the hospital setting and review “Millie’s” medication history to complete full medication reconciliation since Millie probably fell due to poor lighting, neuropathy, and hypoglycemia. In stage 2, students review the demonstration of a mentation assessment for dementia and depression using screening tools (PHQ 2 and Mini-Cog) since Millie developed delirium after her surgery for a fractured hip. In stage 3, the patient Millie is ready to be discharged, and the students review “What Matters” and an introductory discussion of advance care directives with the patient’s caregiver while she is discharged to a skilled nursing facility. In the final stage, Millie is transferred to a skilled nursing facility for rehab. The students learn to use the fall risk assessment Timed Up and Go (TUG) while answering an interactive quiz to identify fall risk hazards, such as handlebars, tables, bed height (too high), no shower chair, no night light, etc. Medical students were also taught interviewing skills to help them communicate with patients struggling with dementia and encourage advanced care discussion for appropriate intervention and referral. 

### Descriptive Statistics

In total, 400 medical students received VR geriatric training, and 40 (10%) completed the knowledge survey and provided demographic information. Thirty-two (80%) participants were non-Hispanic, twenty-six (65%) were female, and thirteen (33%) were male. Twenty-six (65%) participants identified themselves as White. The majority of the participants, 36 (90%), were in the age range of 20–29 years old. (See Table 1). Survey participants were asked to rate their knowledge on the topic of the Age-Friendly Health System and the 4Ms; they were also asked to score on multiple-choice questions specific to what matters, such as medication, mentation, and mobility.

*General Knowledge Pre/Post-Training.* The goal of the study is to compare changes in knowledge towards AFHS before and after the completion of the training. The survey questions evaluated students’ knowledge through a three-point Likert scale with low, moderate, and high responses. Scores were entered in SPSS and the data distribution was not normal according to the Kolmogorov–Smirnov test, *p* < 0.05. The Wilcoxon signed-ranks test was conducted and indicates that post-test ranks were significantly higher than pre-test ranks; z = −3.310, *p* < 0.05 (see Table 2). Twenty-three (58%) participants responded that they would apply the training in their practice. 

*Content-based Knowledge Post-Training.* After the training, the survey also asked multiple-choice questions about what matters, medication, mentation, and mobility. These questions were designed to evaluate the ability of the student to “use” the AFHS in a clinical case. Each question consisted of four correct and one incorrect answer. All responses were analyzed and compared. The comparative analysis of student responses on specific topics showed that a higher number of students provided correct responses on questions related to AFHS, 34 (85%); what matters, 35 (88%); medication, 37 (93%); and mentation,36 (90%) as compared to a question on mobility, 18 (45%). Overall, 13 students (33%) received a perfect score of 100% on each question, and 30 students (75%) scored above 90% on all questions, highlighting a clear understanding of the VR training content (See Table 3). 

## 4. Discussion

Our study aims to demonstrate the process of developing, implementing, and evaluating a virtual reality case to teach medical students. Planning and developing a virtual reality case is fun and engaging; however, learning in a virtual environment has its own set of challenges [13]. One study found that the development and implementation of VR applications are associated with high costs, reduced face-to-face interaction, and a cumbersome design and development process. Another review study found technological challenges and usability issues in VR implementation [14]. Our study also encountered several development and implementation challenges. Learning space for the VR experience of AFHS was limited to a few students for each session. This was due to a limited amount of available VR equipment, including VR laptops with Acadicus access and Oculus headsets. Additionally, faculty training was time-consuming, with a learning curve. Several faculty members preferred teaching in a didactic environment and, even though motivated, struggled through the technological issues in navigating the case. 

Evaluation feedback from the students was positive and demonstrated a significant change in students’ knowledge about the 4Ms of the Age-Friendly Health System. There was a significant improvement in knowledge of the 4Ms of the AFHS. However, these differences in knowledge post-training may be biased due to a low response rate, social desirability, and self-report responses. It is also difficult to estimate if participants remembered the information after completing the case experience. Survey fatigue and participants skipping questions on the knowledge survey were among other limitations. In addition, the VR reality case was uploaded as a YouTube link and distributed during the class. Our survey did not ask questions about the differences in the experience of YouTube video training vs. real-time VR case scenarios. 

Interactive virtual reality is utilized to train medical students on multiple topics to supplement traditional teaching. Future studies should address the development, implementation, and evaluation limitations to evaluate the short-term and long-term impact of training a healthcare workforce through virtual reality case-based scenarios. An in-depth review of the sustainability of virtual education suggests understanding sustainability from a systems approach [15]. The review highlights strategies for evaluating learning content and objectives, evaluation, target learners, innovation, and quality assurance while developing and implementing virtual medical education [15]. 

## 5. Conclusions

This quality improvement project demonstrates the value of VR applications in medical student education programs. Innovative teaching models such as VR have been helpful in student engagement due to their ability to simulate a real-world environment. Medical education is continuously evolving and adapting to new opportunities for student learning. Our study planned, developed, implemented, and evaluated a case-based scenario to teach the 4Ms of the AFHS through VR application scenarios. The curriculum included an innovative way to teach geriatrics to medical students by integrating several components essential for effective and engaging education, including hands-on clinical experience, interprofessional education, and autonomous learning, in a safe, controlled environment. Medical education must keep pace with the relentlessly changing medical practices and the complex health needs of the growing population of older adults.

## Figures and Tables

**Table 1:** Demographics.

Sample Characteristics	# of Students	% of Students
M3/M4	40	
Age		
20–29	36	90%
30–39	4	10%
Gender		
Female	26	65%
Male	13	32%
Not Reported	1	3%
Race		
White	26	65%
African American	2	5%
Asian	10	25%
Unspecified	2	5%
Ethnicity		
Hispanic	7	18%
Non-Hispanic	32	80%
Unspecified	1	3%

**Table 2:** General Knowledge Pre/Post-Training.

(**a**) **(Wilcoxon Signed-Ranks Test).**			
Post-Test Scores-Pre-Test Scores	N	Mean Rank	Sum of Ranks
Negative Ranks	1 ^a^	16.00	16.00
Positive Ranks	18 ^b^	9.67	174.00
Ties	21 ^c^		
Total	40		
^a^. post_training scores < pre_training scores			
^b^. post_training scores > pre_training scores			
^c^. post_training scores = pre_training scores			
(**b**) **(Result of Statistical Test).**			
Post-Test Scores-Pre-Test Scores			
Z	−3.310 ^b^		
Asymp. Sig. (2-tailed)	0.001		
^b^. Based on negative ranks.			

**Table 3:** Participant’s knowledge post-training.

Post-Training Participants’ Knowledge Rating (Success Rate) N = 40	# of Participants with Correct Responses	% of Participants with Correct Responses
Good (80%+)—learned a lot of the presented material	37	93%
High (90%)—learned most of the presented material	30	75%
Excellent (100%)—learned all of the presented material	13	33%

## Data Availability

The data was collected as part of a Federal project and is unavailable due to privacy and ethical restrictions.
